# PENet—a scalable deep-learning model for automated diagnosis of pulmonary embolism using volumetric CT imaging

**DOI:** 10.1038/s41746-020-0266-y

**Published:** 2020-04-24

**Authors:** Shih-Cheng Huang, Tanay Kothari, Imon Banerjee, Chris Chute, Robyn L. Ball, Norah Borus, Andrew Huang, Bhavik N. Patel, Pranav Rajpurkar, Jeremy Irvin, Jared Dunnmon, Joseph Bledsoe, Katie Shpanskaya, Abhay Dhaliwal, Roham Zamanian, Andrew Y. Ng, Matthew P. Lungren

**Affiliations:** 1 Department of Biomedical Data Science, Stanford University, Stanford, CA USA; 2 Center for Artificial Intelligence in Medicine & Imaging, Stanford University, Stanford, CA USA; 3 Department of Computer Science, Stanford University, Stanford, CA USA; 4 Department of Biomedical Informatics, Emory University, Atlanta, GA USA; 5 Department of Radiology, Stanford University, Stanford, CA USA; 6 Department of Emergency Medicine, Intermountain Medical Center, Salt Lake Valley, UT USA; 7 Michigan State University, College of Human Medicine, East Lansing, MI USA; 8 Department of Pulmonary Critical Care Medicine, Stanford University, Stanford, CA USA; 9 Vera Moulton Wall Center for Pulmonary Vascular Disease, Stanford University School of Medicine, Stanford University, Stanford, CA USA

**Keywords:** Cardiovascular diseases, Radiography

## Abstract

Pulmonary embolism (PE) is a life-threatening clinical problem and computed tomography pulmonary angiography (CTPA) is the gold standard for diagnosis. Prompt diagnosis and immediate treatment are critical to avoid high morbidity and mortality rates, yet PE remains among the diagnoses most frequently missed or delayed. In this study, we developed a deep learning model—PENet, to automatically detect PE on volumetric CTPA scans as an end-to-end solution for this purpose. The PENet is a 77-layer 3D convolutional neural network (CNN) pretrained on the Kinetics-600 dataset and fine-tuned on a retrospective CTPA dataset collected from a single academic institution. The PENet model performance was evaluated in detecting PE on data from two different institutions: one as a hold-out dataset from the same institution as the training data and a second collected from an external institution to evaluate model generalizability to an unrelated population dataset. PENet achieved an AUROC of 0.84 [0.82–0.87] on detecting PE on the hold out internal test set and 0.85 [0.81–0.88] on external dataset. PENet also outperformed current state-of-the-art 3D CNN models. The results represent successful application of an end-to-end 3D CNN model for the complex task of PE diagnosis without requiring computationally intensive and time consuming preprocessing and demonstrates sustained performance on data from an external institution. Our model could be applied as a triage tool to automatically identify clinically important PEs allowing for prioritization for diagnostic radiology interpretation and improved care pathways via more efficient diagnosis.

## Introduction

With an estimated 180,000 deaths per year, pulmonary embolism (PE) remains a leading cause of death in the United States.^1^ The definitive diagnosis of PE is made on imaging via computed tomography pulmonary angiography (CTPA).^2^ Prompt recognition of the diagnosis and immediate initiation of therapeutic action (anticoagulation and mechanical thrombectomy) is important because delay in PE diagnosis and treatment is associated with substantially increased morbidity and mortality rates.^3,4^ Unfortunately PE remains among the diagnoses most frequently missed, in part due to lack of radiologist availability, physician fatigue, and diagnostic error. Studies has shown that there can be up to a 13% discrepancy rate between overnight radiologists and daytime faculty.^5–7^ Increasing pressure is placed on hospital systems to provide 24–7 access to advanced imaging and to ensure that the results of urgent findings, such as PE, are rapidly and accurately communicated to the referring clinician.^8,9^ However, providing rapid and accurate diagnostic imaging is increasingly difficult to sustain for many medical systems and radiology providers as utilization has expanded; for example, CTPA usage alone in the emergency setting has increased 27-fold over the past 2 decades.^10,11^

Applications of deep learning have already shown significant promise in medical imaging including chest and extremity X-rays,^12–15^ head CT,^16^ and musculoskeletal magnetic resonance imaging (MRI).^17^ But despite the potential clinical and engineering advantages for utilization of deep learning automated PE classification on CTPA studies, significant development challenges remain when compared to other applications. For example, CTPA examinations are orders of magnitudes larger than most common medical imaging examinations (i.e., chest X-rays or head CT) and PE findings represent only a small fraction of the pixel data relative to the 3D CTPA volume. Further exacerbating this signal-to-noise problem are the extreme inter-image and interclass variance unique to CTPA studies caused by a reliance on timing of intravenous contrast injection protocol and patient compliance with breath holding instructions; the variations in breathing motion and timing of contrast bolus injection lead to artifacts and increased noise.^18^ Lastly, generalization across institutions particularly in the setting of varying CT scanner models and reconstruction methods present another difficult problem in generalization for automated diagnosis.

Despite these challenges, deep learning for automated PE diagnosis on CTPA as an end-to-end solution, if successful, could serve as an excellent clinical use case because (1) PE is a common lethal disease and strategies for rapid, accurate, diagnosis is of high concern to clinicians, patients, payers, and regulatory bodies; (2) CTPA imaging is the most commonly performed imaging examination for PE diagnosis; (3) definitive diagnosis of PE can be made on CT imaging (further diagnostic work-up or pathologic confirmation not needed) which fits well for an supervised learning approach. The emerging application of deep learning models in medical imaging is enabling a collaborative man-machine environment by intelligent prioritization of radiology studies for interpretation to reduce time to diagnosis for critical diseases and identifying abnormalities (i.e., brain hemorrhage on head CT imaging) and motivates the development of new algorithms for other emergent conditions such as PE on CTPA exams.^17,19,20^

The bulk of the early work in automated PE diagnosis has focused on leveraging clinical variables and/or ventilation-perfusion imaging (rather than CTPA) as inputs to run simple artificial neural networks with modest success and limited clinical applicability due to poor generalization.^21–24^ Prior efforts toward automation of PE diagnosis using CTPA have focused on traditional feature engineering methodologies; while the higher performing among these have reported sensitivity as high as 75% for diagnosing PE on CTPA studies, each have significant drawbacks due to the relatively high burden of development and implementation, including manual feature engineering, complex preprocessing adding significant time and infrastructure costs, and a lack of external validation to understand the generalizability and identify overfitting.^25–30^ In contrast, advancements in deep learning possess inherent advantages over prior approaches due, in part, to obviating the need for hand crafted feature engineering and flexibility as an “end-to-end” classification solution.

More recent work has turned toward CTPA imaging PE diagnosis using convolutional neural networks (CNNs); for example, Tajbakhsh et al.^30^ applied 3D CNN on CT imaging to detect PE and relied on extensive preprocessed generated features via segmentation and vessel-alignment as an input for their CNN model. Similarly, Yang et al.^31^ reported a two-stage convolutional neural network with a state-of-the-art 0.75 sensitivity on a small test set (20 patients), however, they subdivided each CTPA into small cubes to evaluate model performance rather than the entire CTPA scan. These approaches, while among the first to address the significant technical challenges of automated PE diagnosis on CTPA, suffer from artificially constrained study conditions and lack a viable “end-to end” solution required for realistic real time clinical application.

The purpose of this work is to develop and evaluate an end-to-end deep learning model capable of detecting PE using the entire volumetric CTPA imaging examination with simultaneous interpretability at the volumetric level that is robust to application on external dataset. If successful this work may lead to applications for timely PE diagnosis, including in resource constrained settings.

## Results

### Model performance

**Fig. 1:**
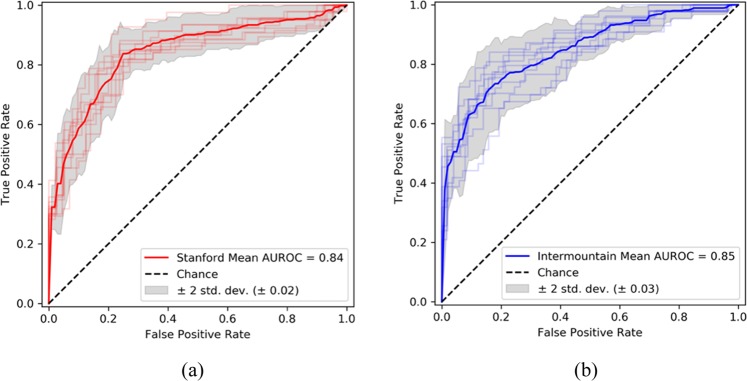
PENet performance on independent test datasets. Receiver operating characteristic curve (ROC) with bootstrap confidence intervals on Stanford internal test set (**a**) and Intermountain external test set (**b**).

The performance of PENet on the internal (Stanford) and external (Intermountain) tests sets are detailed in Table 1. Over the entire Stanford hold-out test set of 169 studies (84 negative and 85 positive), the PENet achieved an AUROC of 0.84 [0.82–0.87] (Fig. 1). For the external validation dataset from Intermountain with 200 studies (106 negative and 94 positive), the PENet model trained only on Stanford dataset, achieved an AUROC of 0.85 [0.81–0.88]. The published overall rate of positive diagnosis of PE on pulmonary CTA varies from study to study but usually ranges between 14 and 22%.^32,33^ To simulate prevalence of PE in the real world, we adopted a new bootstrap testing strategy where we randomly selected within a range of 14–22% of positive cases and the remaining negative cases. In order to test generalizability, we simulated the sampling 100 times and this gave us an AUROC of 0.84 [0.79–0.90] on the Stanford dataset and 0.85 [0.80–0.90] on the Intermountain dataset (Table 1). The accuracy, specificity and sensitivity stayed fairly consistent between the balanced test set and bootstrapped experiment. As expected, the positive predictive value (PPV) and negative predictive value (NPV) were affected by the difference in positive and negative case distribution—low number of true positives (only 14–22%) in the dataset and our choice of a high sensitive operating point (as mentioned in the Discussion) contributes to lower PPV and higher NPV. In the clinical setting, however, optimizing sensitivity of the positive cases is more relevant than PPV (more false positives) since our system will be used as a worklist triage tool and all cases with probabilities higher than the threshold can be further checked and filtered by radiologists. Such human–machine interaction will improve both PPV and NPV value.

**Table 1:** Model performance.

	Internal dataset: Stanford	Internal dataset: Stanford (real prevalence)	External dataset: Intermountain	External dataset: Intermountain (real prevalence)
*Metric*				
Accuracy	0.77 [0.76–0.78]	0.81 [0.80–0.82]	0.78 [0.77–0.78]	0.80 [0.79–0.81]
AUROC	0.84 [0.82–0.87]	0.84 [0.79–0.90]	0.85 [0.81–0.88]	0.85 [0.80–0.90]
Specificity	0.82 [0.81–0.83]	0.82 [0.82–0.83]	0.80 [0.79–0.81]	0.81 [0.80–0.82]
Sensitivity	0.73 [0.72–0.74]	0.75 [0.73–0.77]	0.75 [0.74–0.76]	0.75 [0.73–0.77]
PPV/precision	0.81 [0.80–0.81]	0.47 [0.45–0.48]	0.77 [0.76–0.78]	0.44 [0.43–0.46]
NPV	0.75 [0.74–0.76]	0.94 [0.94–0.95]	0.78 [0.77–0.79]	0.94 [0.94–0.95]

Model performance on the internal test set (Stanford) and external test set (Intermountain) with 95% confidence interval using probability threshold of 0.55 that maximizes both sensitivity and specificity on Stanford validation dataset. Bootstrapping is used to generate prevalence of PE in real world (between 14 and 22%).

### Comparison with state-of-the-art 3D CNN models

As detailed in Table 2, PENet with 24 slices input outperforms ResNet3D, ResNeXt3D and DenseNet3D by a wide margin: 0.04 AUROC higher on the internal test dataset and 0.02 AUROC higher on the external test dataset as compared to the next best performing model. It is important to note that all models converged during the training process. We also compare the effect of pretraining our PENet model with the Kinetics dataset. The no-pretrain version of PENet is trained first by using the same pretrain hyperparameters then the same fine-tune hyperparameters as the standard PENet. Pretraining PENet increases performance by 0.15 AUROC on the internal test set and 0.23 AUROC on the external test set. The current state-of-the-art PE detection model by Yang et al.^31^ reported a sensitivity of 0.75. based on 20 patients, however, these results are incomparable to ours since the authors subdivided each CTPA into small cubes to evaluate model performance rather than the entire CTPA scan. We attempted to test the model developed by Yang on our dataset to produce a comparable result, but the codebase published by the authors is not trivial to reproduce.

**Table 2:** Comparison with state-of-the-art 3D CNN models.

	Internal dataset: Stanford	External dataset: Intermountain
Metric (AUROC)		
PENet—24 slices kinetics pretrained	0.84 [0.82–0.87]	0.85 [0.81–0.88]
PENet no pretraining	0.69 [0.74–0.65]	0.62 [0.57–0.88]
ResNet3D-50 kinetics pretrained	0.78 [0.74–0.81]	0.77 [0.74–0.80]
ResNeXt3D-101 kinetics pretrained	0.80 [0.77–0.82]	0.83 [0.81–0.85]
DenseNet3D-121 kinetics pretrained	0.69 [0.64–0.73]	0.67 [0.63–0.71]

AUROC on the internal test set (Stanford) and external test set (Intermountain) with 95% confidence interval: ResNet3D^47^, ResNeXt3D^45^ and DenseNet3D^46^ were pretrained with Kinetics-600 and finetuned using the Internal dataset using the same training hyperparameters as PENet. PENet outperforms each of these models on both the internal and external test set.

### Clinical utility

**Fig. 2:**
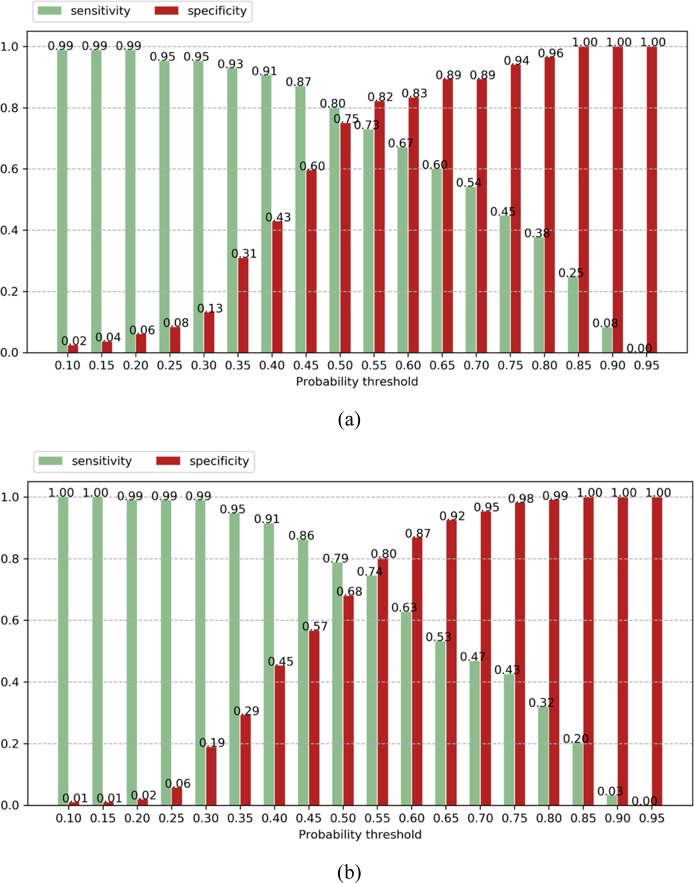
(Sensitivity vs. specificity plot): Sensitivity and specificity across different operating point (probability threshold) with increment of 0.05 on the Stanford internal test set (**a**) and Intermountain external test (**b**).

**Fig. 3:**
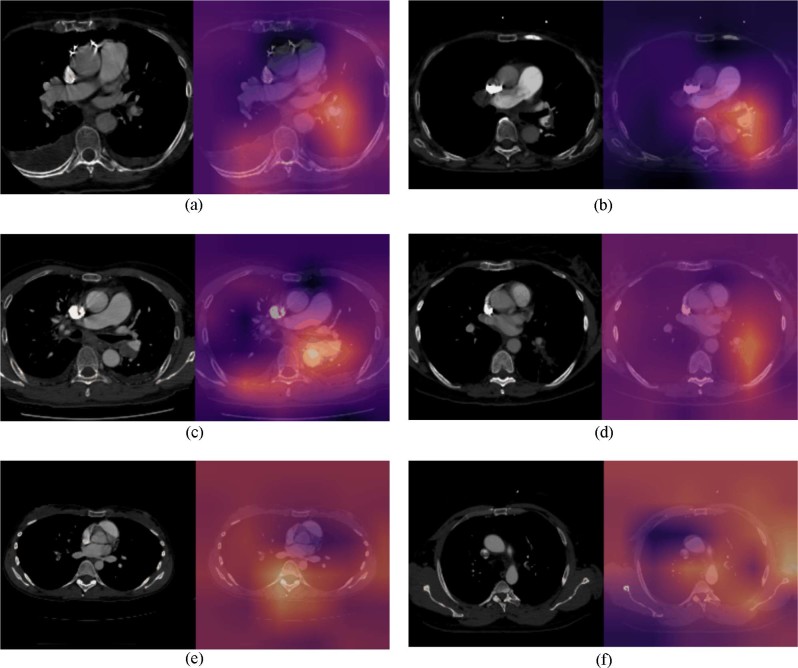
(Class Activation Maps): Class activation map (CAM) representation of true positive (Stanford (**a**) and Intermountain (**b**), false-positive (Stanford (**c**) and Intermountain (**d**) and false-negative samples (Stanford (**e**) and Intermountain (**f**)—axial contrast enhanced CT pulmonary angiogram (left) and CAM inferred by the model overlay with the scan (right). **a** (Stanford test set: true positive): (left) demonstrates a non-occlusive filling defect in a left lower pulmonary artery segmental branch that is correctly localized by the model as seen in the CAM image overlay (right). **b** (Intermountain test set: True Positive): (left) demonstrates a non-occlusive filling defect in a left main pulmonary artery that is correctly localized by the model as seen in the CAM image overlay (right). **c** (Stanford test set: false positive): left) demonstrates a large left hilar node adjacent to the pulmonary artery that is incorrectly labeled as PE by the model as seen in the CAM image overlay (right). **d** (Intermountain test set: false positive): (left) demonstrates an enlarged unopacfied left lower lobe pulmonary vein invaded by tumor that is incorrectly labeled as PE by the model as seen in the CAM image overlay (right). **e** (Stanford test set: false negative): (left) Pulmonary embolism in right middle lobe segmental branch that is missed by the model as seen in the CAM image overlay (right). **f** (Intermountain test set: False negative): (left) Pulmonary embolism in left upper lobe segmental branch that is missed by the model as seen in the CAM image overlay (right).

In order to understand the clinical utility of the pretrained PENet model, Fig. 2 shows the sensitivity and specificity of our model as a bar graph under different operating points. In this study, we set our operating point at a threshold that maximizes both sensitivity and specificity on the Stanford validation set to dichotomize the model’s predictions with *P* ≥ 0.55 (Table 1). This threshold allows our model to achieve a sensitivity of 0.73 [0.72–0.74] and specificity of 0.82 [0.81–0.83] for the Stanford test data, as well as sensitivity of 0.75 [0.74–0.76] and specificity of 0.80 [0.79–0.81] for the Intermountain dataset. While a standard probability threshold of 0.5 results in 0.80 sensitivity and 0.75 specificity for Stanford, as well as 0.79 sensitivity and 0.68 specificity for Intermountain. Applications in clinical settings, however, are usually tuned to maximize sensitivity in order to minimize the false negative rate. If we use an operating point of 0.40 to increase sensitivity, our model can achieve a sensitivity of 0.91 for both the internal and external test set but sacrifices specificity to 0.43 and 0.45, respectively and results more false-positive cases (Fig. 2). We can further improve PENet’s sensitivity with the cost of higher false-positive rates. In Fig. 3, we represent the Class activation map (CAM) for Stanford and Intermountain sample data. The CAMs not only add explain-ability to the model and localization of the PE but also help to understand the error-rate through visualization. For both dataset, we also showed CAMs for false-positive and false-negative samples which reflect the fact that the model is confused with mirroring appearance of difference disease for false-positive cases.

## Discussion

The purpose of this work was to develop and evaluate an end-to-end deep learning model capable of detecting a PE using the entire volumetric CTPA imaging examination with simultaneous interpretability at the volumetric level that is robust to application on external dataset. Our model achieved AUROC of 0.84 on automatically detecting PE on the hold out test set from Stanford and AUROC of 0.85 on external Intermountain datasets. The high performance of our model on the external dataset which had both different slice thickness and scanner manufacturer type, indicating that the model was robust to the differences between datasets and not, as has been seen in prior work, failed by learning non-clinical features as has been demonstrated in other cross-institutional work.^34^ In contrast to state-of-the-art, our results demonstrate feasibility for study-level, robust, and interpretable diagnosis including sustained cross-institutional AUROC performance on an external dataset. Thus, this work supports that successful application of deep learning to the diagnosis of a difficult radiologic finding such as PE on complex volumetric imaging in CTPA is possible, and can generalize on data from an external institution despite that the external institution used a different CT scanner and imaging protocol settings. The proposed model also outperformed state-of-the-art 3D CNN models—ResNeXt3D and DenseNet3D for the same task.

To summarize, the core contributions of this work is: (1) development of a scalable (and open-source) 3-D convolution model for diagnose of PE on CT imaging that has been tested on patient data from two hospital systems (Stanford and Intermountain); (2) an outcome-labeled imaging dataset composed of CTPA studies to enable others to reproduce our methods, validate results, and to ideally allow for further innovation (3) an end-to-end model that can ingest volumetric CTPA imaging studies without pre-processing or feature engineering. Ultimately clinical integration may aid in prioritizing positive studies by sorting CTPA studies sensitivity for timely diagnosis of this important disease including in settings where radiological expertize is limited.

Radiologists all over the world are reading rising numbers of imaging studies with ever increasing complexity amidst ongoing physician shortages; this trend affects both medically underserved populations as well as modern healthcare systems.^35^ Further, even experienced radiologists are subject to human limitations, including fatigue, perceptual biases, and cognitive biases, all of which lead to errors.^36^ These forces strongly motivate leveraging deep learning to perform tasks in medical imaging, and as demonstrated here, could be used to detect PE automatically via worklist prioritization, allowing the sickest patients to receive quicker diagnoses treatment. In this scenario, the studies detected as abnormal by the model could be moved ahead in the image interpretation workflow to aid the radiologist, while the examinations identified as confidently normal could be automatically assigned a preliminary reading of “normal” allowing both triage for lower priority on a worklist. Our model may best suited in identifying medically important PEs as a triage tool.^35,37^ We engineered the model such that it could automatically generate an interpretable prediction value to provide an objective quantification of PE positive risk to help inform ideal thresholds for clinical application in a diagnostic workflow. For example, we could set probability thresholds of 0.3 allowing the model to be more sensitive while sacrificing accuracy and increasing false positives (see Fig. 2), something that would be tolerated in a worklist triage tool that functions to reorder the radiologist’s studies to be read by order of likelihood of PE (rather than the current model of by time acquired). Rapid preliminary results by the model can be conveyed to the ordering provider (and patient) which could improve disposition in other areas of the healthcare system (i.e., discharged from the ED more quickly). Further, the objective prediction score output may allow earlier treatment for PE in patients that return a high positive PE prediction score, which may improve clinical outcomes for those patients as it may allow for rapid/early treatment.^38^ More studies are necessary to evaluate the optimal integration of this model and other deep learning models in the clinical setting.

This study had important limitations. This was a retrospective study design with well described shortcomings. The deep learning model described was developed and trained on data from one large academic institution and although performance was sustained in a new dataset from another institution, universal generalizable performance is not known. We optimized development of the model to focus on clinically important PE as an emergent triage tool in keeping with clinical definitions for recommended treatment and cases of chronic or subsegmental PE were not included.^35^ In a clinical environment, CTPA examinations can also be used to evaluate for other important diagnoses not just for PE, though this is not common practice, and our model as designed would not identify other important pathologies. Lastly, We did not perform an analysis on specific artifacts in either the internal or external dataset (this is difficult to quantify as a label on what constitutes artifact in terms of limiting diagnostic performance) as this was not a primary label for the dataset. However, we used retrospective datasets from both institutes (Stanford and Intermountain) and did not exclude studies for the purpose of motion artifact (used clinical labels). Therefore, we believe that our results are, while less than perfect, are more representative of routine clinical practice than had we intentionally removed cases with motion or bolus artifacts.

In conclusion, we developed a predictive deep learning model capable of detecting PE on CTPA with validation on data from an outside institution. The sustained performance on external validation data supports potential applicability of this technology to improve healthcare delivery for patients being evaluated for PE with CT. Further studies are necessary to determine patient outcomes and model performance in a prospective clinical setting.

## Methods

### Internal dataset

We retrospectively collected 1797 CTPA studies from 1773 unique patients performed under the PE Protocol (LightSpeed QXi, GE Healthcare, Milwaukee, USA) at Stanford University Medical Center (SMC) (Table 3). These studies were pulled from local picture archiving and communication system (PACS) servers and anonymized in compliance with internally defined Health Insurance Portability and Accountability Act (HIPAA) guidelines. Axial CT series with a slice thickness of 1.25 mm were extracted for the development of our algorithm. These studies were split into a training set (1461 studies from 1414 patients), a validation set (167 studies from 162 patients) and a hold-out test set (169 studies from 163 patients). To generate the validation and test sets, stratified random sampling was used to ensure that there was an equal number of positive and negative cases. Care was taken to ensure that there was no patient overlap between training, validation, and test sets.

**Table 3:** Data characteristics of the internal (SMC) and external (Intermountain) dataset.

	Overall	Train	Validation	Test	External test (intermountain)
Number of studies	1797	1461	167	169	200
Median age (IQR)	66.14 (53.24–82.40)	66.13 (53.14–82.95)	64.10 (50.88–78.38)	67.24 (56.62–82.76)	55.3 (42.0–69.5)
Number of patients (Female %)	1773 (57.07%)	1414 (56.64%)	162 (67.36%)	163 (52.08%)	198 (58.5%)
Median number of slices (IQR)	386 (134)	385 (136)	388 (132)	388 (139)	324
Number of positive PE	655	488	82	85	94
Number of negative PE	1142	973	85	84	106

The internal SMC dataset was divided into training, validation and test. The training set was used to optimize model parameters and the validation set was used to select the best model and operating points. The hold-out test set was used to evaluate the model’s performance. The external Intermountain dataset was used solely for evaluation.

### External validation

In order to evaluate the generalizability across institutions of the model performance for PE detection, 200 CTPA studies from 198 patients performed under the PE protocol (Aquilion Toshiba Medical Systems, Otawara, Japan) were collected from Intermountain healthcare system. This external dataset was not available during the training process and was only used to evaluate the model performance. Axial CT series with a slice thickness of 2 mm were extracted. Stratified random sampling technique is used to create the external test set. Table 3 describes the datasets and patient demographics for each data partition.

### Annotations and image preprocessing

For the entire cohort (internal and external) two board-certified radiologist manually reviewed each study (scans and radiology reports). One radiologist BP has 8 years of experience and the other ML has 10 years of experience in clinical radiology practice. Interrater reliability was estimated as Cohen’s Kappa Score and the raters were highly consistent for determining PE present with kappa scores of 0.959. The senior radiologist resolved all conflicting cases manually for preparing the ground truth labels. We used standard descriptions of PE to label PE negative, PE positive and subsegmental-only PE positive studies, with slight modifications to account for anatomic variations and the orientation of vessels in the transverse plane on CT scans.^39^ Particularly, subsegmental only PE was defined as the location of the largest defect at the subsegmental level on a spiral CT allowing a satisfactory visualization of all pulmonary arteries at the segmental level or higher. Subsegmental only PE is felt to be of questionable clinical value, so we removed all subsegmental only PE studies from our dataset.^40^ Training data were labeled on a slice level for the presence/absence of a PE. Before feeding into the model, examinations were extracted from Digital Imaging and Communications in Medicine (DICOM) format and scaled to 512 × 512 pixels. The entire series of *N* slices was saved as a *N* × 512 × 512 array.

### PENet architecture

**Fig. 4:**
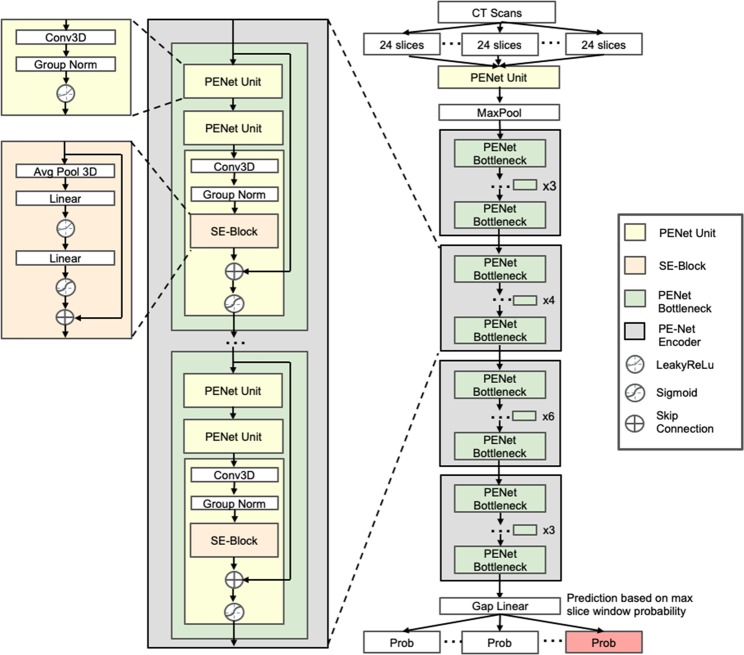
PENet architecture used in this study. PENet is built using four architectural units: the PENet unit, Squeeze-and-Excitation block, the PE-Net bottleneck, and the PE-Net encoder. Each building block in the network is color-coded.

PENet is a 3D convolutional neural network that aims to detect the PE in a series of slices from a CTPA study (Fig. 4). The use of 3D convolutions allows the network to use information from multiple slices of an exam in making each prediction. That is, with 2D convolutions each slice would be considered independently, whereas 3D convolutions aggregate information from many consecutive slices. This is especially relevant in diagnosing PE, where the abnormality rarely occupies just a single slice. The model that we developed, the PENet, is built using four architectural units: the PENet unit, Squeeze-and-Excitation block, the PE-Net bottleneck and the PE-Net encoder^41^ (Supplementary Table 1). The PENet unit is meant to process 3D input data, using a 3D CNN followed by group normalization and activated by LeakyReLu.^42^ The Squeeze-and-Excitation block (SE-block) serves to model the interdependencies between channels of the input and adaptively recalibrates channel-wise features. A PENet bottleneck is built using three PENet units, with a SE-block inserted after the group normalization layer of the last PENet unit. A skip-connection is also applied between the PENet bottleneck input and the SE-block output. Multiple PENet bottlenecks, ranging from three to six, join in sequence to build the PENet encoder. Our final model consists of an individual PENet Unit, following by four PENet encoders and GapLinear activation. The depth of the network was chosen via cross-validation on the training data: shallower networks were not able to model the complexity of the dataset, whereas deeper networks showed lower performance on a held-out validation set due to overfitting.

### Network training strategy

The PENet was pretrained on the Kinetics-600 dataset,^43^ after which we replace the 600-way softmax layer with a single-output linear layer and sigmoid activation. To accommodate large input sizes with limited GPU memory requiring small batch sizes, we replaced batch normalization with group normalization throughout.^44^ For regularization, we applied L2 regularization of 1 × 10^−3^ to all learnable parameters. For the loss function, we used binary cross-entropy focal loss to counteract class imbalance and focus training on difficult examples (despite the balanced number of positive and negative cases, the window-level dataset is heavily skewed towards negative examples). The optimized hyper-parameters for PENet can be found in Supplementary Table 2.

Given the small PE targets relative to the input volume, PENet takes in a sliding window of 24 slices at a time instead of the entire volumetric CT scan. Using this sliding window approach increases the proportion of the target PE relative to the input which ultimately escalates the model optimization and reduce the requirement of computational resources. The choice of 24 slices is determined through experimentation on the validation data (Table 4); smaller number of input slices does not provide enough information for the model to learn while too many input slices makes PE hard to detect. Before training, all slices consisting of raw Hounsfield Units are clipped to the range [−1000, 900] and zero-centered. During training we resized each CT slice to 224 × 224 for computational efficiency, randomly cropped to 192 × 192 along the height and width axes, rotated up to 15°, and jittered up to 8 slices along the depth dimension for data augmentation. After passing each batch, the gradient of the loss was computed on the batch, and PENet’s parameters were adjusted in the opposite direction of the gradient. We used stochastic gradient descent with momentum of 0.9 and an initial learning rate of 0.1 for the randomly initialized weights and 0.01 for the pretrained weights. We labeled a window as positive if it contains at least 4 abnormal slices, and we up-sampled positive windows at a rate of 35%. Since the smallest PE still takes up 4 slices of CT, the number of abnormal slices for a positive window is chosen via cross validation, ranging from 1 slice to 4 slices. The upper limit of our cross-validation range is determined by the number of slices the smallest PEs in our dataset take up. During the training process, we realize that if we chose more than this upper limit, some positive studies will contain no positive window which may introduce more outlier. For the learning rate schedule, we adopted a linear warmup for 10k iterations, followed by cosine annealing for 600k iterations. Additionally, we delayed the learning rate schedule by 10k iterations for the pretrained weights. The model parameters were saved after every epoch and the model with the highest AUROC on the validation set was chosen for evaluation on the test set.

**Table 4:** Input slice number experimentation.

	Internal dataset: Stanford	External dataset: Intermountain
Metric (AUROC)		
PENet—1 slice	0.48 [0.45–0.51]	0.51 [0.47–0.54]
PENet—6 slices	0.57 [0.53–0.60]	0.58 [0.55–0.59]
PENet—12 slices	0.74 [0.70–0.77]	0.69 [0.67–0.72]
PENet—24 slices	0.84 [0.82–0.87]	0.85 [0.81–0.88]
PENet—48 slices	0.80 [0.77–0.83]	0.83 [0.76–0.86]

AUROC on the internal test set (Stanford) and external test set (Intermountain) with 95% confidence interval: smaller input slice number does not provide enough structural information to learn while too many input slices makes pulmonary embolism hard to detect.

In order to setup a benchmark for this task, we compare PENet to several common 3D CNN architectures in Table 2. This includes the current state-of-the-art model architecture for Kinetics dataset, ResNeXt3D-101,^45^ as well as memory efficient DenseNet3D-121^46^ and the classic ResNet3D-50.^47^ All of these models are pretrained on the Kinetics dataset then fine-tuned on the internal PE dataset until convergence (exactly like PENet).^48^ A learning rate finder^49^ described by Smith et al. is used to find the optimal learning rate for each of the model architectures mentioned above.

### Test strategy

We sequentially sampled 24-slice windows from each study and passed it through the model to get a window-level prediction. We took the maximum window-level prediction as the series-level prediction. Thus, the series was predicted as PE positive, if the model predicted any one of the windows as positive. This method is intuitive because PE often resides in a few slices of CT scans, therefore one sliding window that is predicted with PE should represent the entire CTPA series. The same testing strategies that were applied on the Stanford test data were used on the Intermountain dataset to ensure consistency between evaluations.

### Interpretation of the model prediction

To ensure interpretability, we identified locations in the scan that contributed most to the classification using CAMs. We have implemented the Class Activation Mapping based on the descriptions from Zhou et al.^50^ Briefly, the authors used a trained CNN classifier to localize the input image by using CAM. After running the CTPA study thought PENet, the CAMs from the final convolutional layer for the input window are extracted. The discriminative image regions used by the model to classify positive PE candidates is computed by taking the global average pooling on all 2048 output features from the last convolutional layer with weights from the fully connected layer. This is then mapped to a color scheme and up sampled and overlaid with the original input slices. Using the weights from the final layer, more predictive features appear brighter, and thus the brightest areas of the heatmap are regions that influence the model prediction the most.

https://www.youtube.com/watch?v=ZdOabYt4Cjo

### Statistical methods

The comprehensive evaluation of the performance of the model on the test sets included area under the receiver operating characteristic curve (AUROC), sensitivity, specificity, accuracy, PPV, and NPV. The predicted probability threshold for returning a positive finding was determined on validation set, ensuring a high sensitivity for PEs while maintaining a reasonable specificity for subsegmental PEs. To measure the variability in these estimates, we calculated 95% DeLong CIs for the AUROC of the model, and 95% Wilson score CIs for sensitivity, specificity, accuracy, PPV, and NPV at each operating point. In addition, to better understand the performance of the algorithm in diagnosing PEs, we calculated these performance metrics and confusion matrices for the entire dataset.

## Supplementary information

Supplementary Information

Reporting-Summary

## Data Availability

The datasets generated and analyzed during the study are not currently publicly available due to HIPAA compliance agreement but are available from the corresponding author on reasonable request. We plan a full open-source release of all data and trained model in summer of 2020.

## Code availability

All the code used for this study can be found at https://github.com/marshuang80/PENet.
