# Author Correction: PENet—a scalable deep-learning model for automated diagnosis of pulmonary embolism using volumetric CT imaging

**DOI:** 10.1038/s41746-020-00310-6

**Published:** 2020-07-28

**Authors:** Shih-Cheng Huang, Tanay Kothari, Imon Banerjee, Chris Chute, Robyn L. Ball, Norah Borus, Andrew Huang, Bhavik N. Patel, Pranav Rajpurkar, Jeremy Irvin, Jared Dunnmon, Joseph Bledsoe, Katie Shpanskaya, Abhay Dhaliwal, Roham Zamanian, Andrew Y. Ng, Matthew P. Lungren

**Affiliations:** 1 Department of Biomedical Data Science, Stanford University, Stanford, CA USA; 2 Center for Artificial Intelligence in Medicine & Imaging, Stanford University, Stanford, CA USA; 3 Department of Computer Science, Stanford University, Stanford, CA USA; 4 Department of Biomedical Informatics, Emory University, Atlanta, GA USA; 5 Department of Radiology, Stanford University, Stanford, CA USA; 6 Department of Emergency Medicine, Intermountain Medical Center, Salt Lake Valley, UT USA; 7 Michigan State University, College of Human Medicine, East Lansing, MI USA; 8 Department of Pulmonary Critical Care Medicine, Stanford University, Stanford, CA USA; 9 Vera Moulton Wall Center for Pulmonary Vascular Disease, Stanford University School of Medicine, Stanford University, Stanford, CA USA

**Keywords:** Cardiovascular diseases, Radiography

Correction to: *npj Digital Medicine*10.1038/s41746-020-0266-y, published online 24 April 2020

The original version of the published Article did not acknowledge the passing of the sixth Author, Norah Borus. To improve clarity, the Author contributions have been updated to include the following: Norah Borus was not able to review the manuscript prior to submission but she was integral to the concept and design phase of the project and contributed significantly to the codebase. Norah Borus passed away on 14th June 2019. Additionally, the second sentence in the second paragraph and the sixth sentence of the penultimate paragraph of the Discussion have been edited for clarity. The HTML and PDF versions of the Article have been corrected.

