# Supplementary material for: PENet—a scalable deep-learning model for automated diagnosis of pulmonary embolism using volumetric CT imaging
Source: NPJ Digit Med. 2020 Apr 24;3:61. doi: 10.1038/s41746-020-0266-y (PMC7181770; doi:10.1038/s41746-020-0266-y)
Supplement: Supplementary file 1 — Supplementary Information [file 41746_2020_266_MOESM1_ESM.pdf]

Supplementary Information

Supplementary Table 1. Table shows the core ingredients of the PENet architecture

**Core ingredients of the network**	**Details**
Total number of learning parameters	28,398,705
PE-Net Encoder	4
PE-Net Bottleneck	16 (3 + 4 + 6 + 3)
SE-Block	16
PE-Net Unit	32 without SE, 15 with SE
Input PE-Net unit, not in PE-Net Encoder	Kernel size = 7, Stride = (1,2,2), Padding=(3,3,3)
CNN of first PE-Net unit in PE-Net Encoder	Kernel size = 1, Stride = 1, Padding = None
CNN of second PE-Net unit in PE-Net Encoder	Kernel size = 3, Stride = 1, Padding = 1
CNN of third PE-Net unit in PE-Net Encoder (With SE-block)	Kernel size = 1, Stride = 1, Padding = None

Supplementary Table 2: Optimized hyper-parameters for PENet

Parameters	Learning rate	Weight decay	Decay step	Batch size	Num epochs	Optimizer	Momentum	SDG dampening
Value	1e-2	1e-3	600000	16	50	SDG	0.9	0.9
